# NOD1/RIP2 Pathway Promotes Glioma Progression Through Microglial M2 Polarization

**DOI:** 10.1111/cns.70674

**Published:** 2025-12-09

**Authors:** Yuxue Luan, Miao Yu, Haimo Zhang, Xiaozu Zhang, Peilun Xiao, Fenghua Zhou, Tianyu Zhao, Xiaoli Wang, Xizhen Wang

**Affiliations:** ^1^ School of Medical Imaging Shandong Second Medical University Weifang China; ^2^ Department of Anatomy, School of Basic Medicine Shandong Second Medical University Weifang China; ^3^ Pathology Department Affiliated Hospital of Shandong Second Medical University Weifang China; ^4^ Medical Imaging Center Affiliated Hospital of Shandong Second Medical University Weifang China

**Keywords:** glioma, microglia, ML130, NOD1/RIP2, polarization

## Abstract

**Background and Aim:**

Glioma is a highly aggressive malignancy of the central nervous system with a poor prognosis. The nucleoside‐binding oligomerization domain‐containing protein 1 (NOD1)/receptor‐interacting protein 2 (RIP2) pathway is involved in various inflammatory responses and is closely associated with microglial polarization. Microglial M2 polarization alters the glioma microenvironment, promoting tumor growth. This study aimed to investigate the role of the NOD1/RIP2 pathway in glioma progression.

**Methods:**

We explored the mechanism of NOD1/RIP2 in glioma progression through bioinformatics analysis, clinical sample evaluation, and in vivo and in vitro experiments. Bioinformatics analysis was conducted to assess NOD1 expression in glioma tissues. Multiparameter MRI and histologic analyses were performed on human tissues, and the correlation between the relative apparent diffusion coefficient (rADC) and NOD1 expression was analyzed. C6 and U251 glioma cells were treated with ML130, a NOD1 inhibitor, and assessed using 5‐ethynyl‐2′‐deoxyuridine (EdU), plate cloning, Transwell, and wound healing assays. Key molecules of the NOD1/RIP2 pathway were examined through immunofluorescence and Western blotting. Quantitative real‐time polymerase chain reaction (qRT‐PCR) was used to detect Arg1 and CD206 expression in BV2 mouse microglia cultured with C6‐derived conditioned medium (CM). The changes in glioma cell biologic behavior were assessed using C6/BV2‐derived CM through EdU, wound healing, and Transwell assays. Finally, the role and mechanism of NOD1 in glioma growth were evaluated using a rat glioma model.

**Results:**

Bioinformatics analysis showed that NOD1 was highly expressed in glioma tissues and strongly correlated with glioma grade. Human brain glioma samples exhibited increased expression of NOD1, RIP2, Iba1, interleukin‐1β, and CD206, with higher expression in high‐grade gliomas compared to low‐grade gliomas. The NOD1 expression was negatively correlated with rADC values. Treatment with ML130 inhibited glioma cell proliferation, migration, and invasion while reducing NOD1 and RIP2 expression. The expression levels of Arg1 and CD206 in BV2 cells cultured with C6‐derived CM decreased in both ML130 and GSK‐583 groups, while the expression levels increased in the ie‐DAP group. Compared with the control group, the expressions of Arg1 and CD206 in microglia in the GSK‐583 + ML130 and GSK‐583 + ie‐DAP groups were inhibited, and the inhibitory effect in the GSK‐583 + ML130 group was more significant. Furthermore, after culturing with C6/BV2 derived CM, the activity of C6 cells was significantly inhibited in the ML130 group, while the activity increased in the ie‐DAP group. The activity of C6 cells in the GSK‐583 + ML130 and GSK‐583 + ie‐DAP groups was significantly decreased, and this effect was more pronounced in the GSK‐583 + ML130 group. ML130‐treated glioma‐bearing rats exhibited reduced tumor growth, suppressed NOD1/RIP2 pathway activation, and inhibited microglial M2 polarization. However, the results observed in the ie‐DAP group were opposite.

**Conclusions:**

NOD1 is an effective predictor of preoperative glioma grade and prognosis. It facilitates glioma progression by promoting microglial M2 polarization through the NOD1/RIP2 pathway.

## Graphical abstract

NOD1 is an effective predictor of preoperative glioma grade and prognosis. It facilitates glioma progression by promoting microglial M2 polarization via the NOD1/RIP2 pathway.

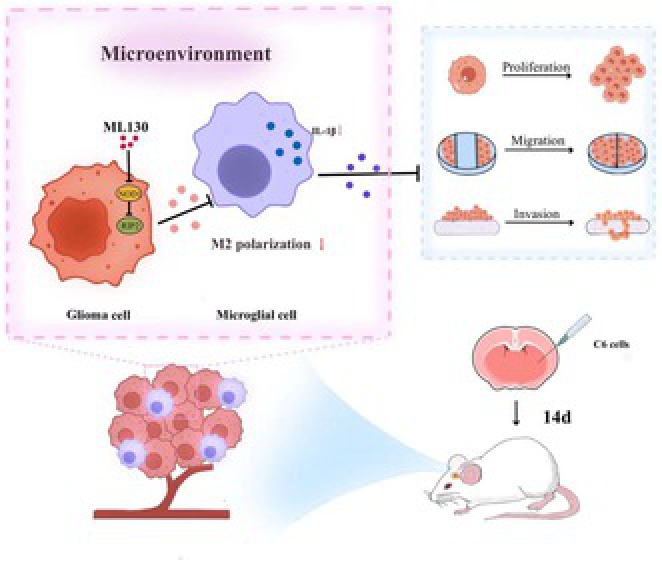

## 1 Introduction

Glioma is a primary malignant tumor of the central nervous system, accounting for more than 80% of all brain malignancies [1]. Based on malignancy, grades I and II are low‐grade gliomas, while grades III and IV are high‐grade gliomas [2]. Glioblastoma (GBM, grade IV) is the most aggressive glioma type [3], characterized by high tumor heterogeneity and immune evasion, with an average survival of < 17 months [4, 5].

Although immunotherapy has been effective against various cancers, including lung cancer [6] and melanoma [7], its efficacy in glioma remains limited due to the unique glioma microenvironment, which includes the blood–brain barrier, an immunosuppressive milieu, and so on [8, 9]. As intrinsic immune cells of the brain, microglia have gained attention for their role in the tumor microenvironment [10]. Microglia exhibit 2 polarization states: M1 (pro‐inflammatory) and M2 (anti‐inflammatory), depending on the stimulation of the microenvironment [11]. M1‐type microglia have antitumor properties and can secrete pro‐inflammatory factors to combat tumors [12]. Conversely, M2‐type microglia can promote tumor growth and immune invasion, facilitating tumor immune escape [13, 14]. Therefore, inhibiting microglial M2 polarization is a key focus in glioma treatment.

Nucleotide‐binding oligomerization domain‐containing protein 1 (NOD1), an intracellular pattern recognition receptor, triggers innate immune responses by recognizing specific pathogen‐associated molecular patterns [15, 16]. NOD1 has been implicated in various inflammatory disorders and is gaining attention for its role in cancer [17, 18]. Its high expression is closely associated with increased tumor malignancy and poorer prognosis, especially in certain solid tumors [19, 20]. Research has demonstrated that NOD1 activates inflammatory pathways such as nuclear factor kappa beta [21] and reactive oxygen species [22] through receptor‐interacting protein 2 (RIP2), influencing immune responses and cell proliferation in tumors [23]. Although macrophages can modulate tumor growth and invasion by NOD1 activation [24], the effect of the NOD1/RIP2 pathway on microglial polarization and glioma progression remains unclear.

This study confirmed that the NOD1/RIP2 pathway accelerated glioma progression by promoting microglial M2 polarization. The human glioma tissues were pathologically stained to detect the expression of the NOD1 protein. In vitro experiments were conducted to examine NOD1 and RIP2 expression in glioma cells treated with ML130, an NOD1 inhibitor, to investigate how the NOD1/RIP2 pathway affects glioma progression. BV2 microglia cells were cocultured with C6‐derived conditioned medium (CM) to observe the polarization states of BV2 cells and evaluate the effect of NOD1 expression on microglial M2 polarization. C6 cells were cocultured with C6/BV2‐derived CM to study the impact of microglial M2 polarization on glioma progression. T2‐weighted imaging (T2WI) MRI combined with various histologic techniques were used in in vivo experiments to investigate how the NOD1/RIP2 pathway affects microglial M2 polarization and glioma progression. This study aimed to highlight the role of the NOD1/RIP2 pathway in glioma progression, providing a theoretical basis for novel glioma treatments.

## 2 Materials and Methods

### 2.1 Clinical Tissue Samples

Patients with primary glioma who had not received preoperative treatment and those requiring surgical decompression were enrolled between January 2021 and December 2023 at the Department of Neurosurgery of Weifang People's Hospital. A total of 57 cases of cerebral glioma tissues (17 cases of low‐grade gliomas and 40 cases of high‐grade gliomas) and six cases of cerebral tissues from traumatic decompression were obtained. This study adhered to the Declaration of Helsinki. All patients provided informed consent, and glioma specimens were confirmed by histopathologic analysis. Preoperative imaging data, including T2WI, T1 contrast‐enhanced imaging (T1CE), and diffusion‐weighted imaging (DWI), were collected. The apparent diffusion coefficient (ADC) of the DWI sequence was measured using MicroDicom software. The experiment was approved by the Medical Ethics Committee of Shandong Second Medical University (approval number: 2022YX048).

### 2.2 Bioinformatics Analysis

Clinicopathologic characteristics of 701 tumor samples and five unpaired normal samples from the Chinese Glioma Genome Atlas (CGGA) database were obtained for gene expression analysis. The specific data is downloaded from the GDC data portal, and the STAR process is used to standardize the data preprocessing. Data visualization and statistical processing were performed using the R language.

### 2.3 Cell Culture

C6 rat glioma cells were purchased from the Shanghai Cell Bank of the Chinese Academy of Sciences, and BV2 mouse microglia cells and U251 human glioma cells were donated by Shandong Unspiral Biological Company. All cell lines were cultured in a humidified incubator with 5% CO_2_ at 37°C using a high‐glucose Dulbecco's modified Eagle medium (Gibco, Rockville, MD, USA) supplemented with 10% fetal bovine serum (Cytiva, DE, USA) and antibiotics (100 U/mL penicillin and 100 mg/mL streptomycin; Gibco).

### 2.4 Cell Viability Assay

The cellular viability of C6 and U251 cell lines was determined using the cell counting kit‐8 (CCK‐8, 40203E; Yeasen, Wuhan, China). The cells (5 × 10^3^ cells/well) were inoculated in 96‐well plates and treated with ML130 (0, 10, 20, 40, 60, and 80 μM) [25], GSK‐583 (0, 5, 10, and 20 μM), or ie‐GAP (0, 15, 30, 60, 90, and 120 μM) (dissolved in dimethyl sulfoxide [DMSO]; MCE, NJ, USA) for 24 h. After adding the CCK‐8 reagent and culturing for a further hour, the absorbance at 450 nm was measured.

### 2.5 Plate Cloning Experiment

The cells (5 × 10^2^ cells/well) were cultured in a complete medium containing ML130 (0, 20, 40, and 80 μM) and the concentration of ML130 was determined by CCK8 results. Following a 14‐day culture period, the cells were stained with 1% crystal violet, fixed with 4% paraformaldehyde, and counted using a microscope.

### 2.6 EdU Assay

Cell proliferation was assessed using the Click‐iT EdU‐594 Cell Proliferation Assay Kit (G1603; Servicebio, Wuhan, China). The glioma cells (5 × 10^3^ cells/well) were inoculated into 96‐well plates and treated with ML130 or ie‐GAP for 24 h. EdU was added for 2 h at 37°C. A pre‐prepared click reaction solution was added and incubated for 30 min. The nuclei were stained with Hoechst 33342, and images were captured using a microscope (BX 51; Olympus, Japan).

### 2.7 Wound Healing Assay

Six‐well plates were used to cultivate the glioma cells (3 × 10^5^ cells/well). Streaks were created using a pipette tip, and cells were treated with a serum‐free medium containing ML130 or ie‐GAP. Streak closure was measured at 0, 24, and 48 h to calculate cell mobility.

### 2.8 Cell Invasion Assay

Transwell chambers (8‐μm pore size) coated with collagen (40184; Yeasen) and solidified for 2 h at 37°C. The glioma cells (2 × 10^4^ cells/well) were inoculated in the upper chamber and treated with a serum‐free medium containing ML130 or ie‐GAP. A medium containing 20% serum was concurrently added to the lower chamber. After 24 h, the cells were examined under a microscope after being fixed with 4% paraformaldehyde and stained with crystal violet.

### 2.9 Immunofluorescence Staining

The glioma cells were treated with various concentrations of ML130 for 24 h. After paraformaldehyde fixation, permeabilization with 0.3% Triton‐X, and blocking with 10% bovine serum albumin, the cells were incubated overnight at 4°C with primary antibodies against NOD1 (DF6378, 1:200; Affinity, China) and RIP2 (DF6967, 1:200; Affinity). The next day, the cells were incubated at 37°C with a rabbit anti‐goat antibody conjugated with Alexa Fluor 488 (ZF0511; ZSGB‐Bio, China). A fluorescent microscope was used to take pictures after labeling the nuclei with DAPI.

### 2.10 Preparation of C6/BV2 CM

The C6 glioma cells were divided into ML130/GSK‐583/ie‐GAP and control groups and cultured with ML130 (40 μM)/GSK‐583 (10 μM)/ie‐GAP (30 μM) and DMSO for 24 h, respectively, followed by culture in a complete medium for another 24 h. Then, C6‐CM was obtained. The BV2 cells were cultured with C6‐derived CM for 24 h. Then, the BV2/C6‐CM and BV2 cells were collected by centrifugation. The BV2 cells were analyzed using qRT‐PCR to detect the expression of anti‐arginase‐1 (Arg1) and CD206. EdU assay, wound healing assay, and Transwell assay were performed to evaluate biologic changes in the expression. The C6 cells cultured with C6/BV2‐CM were assessed for 24 h for proliferation, migration, and invasion capabilities.

### 2.11 qRT‐PCR Analysis

Total cellular RNA was extracted using the Total RNA Extraction Kit (19221; Yeasen), reverse transcribed to cDNA, and subjected to real‐time PCR using the PCR Kit (11201; Yeasen). Relative mRNA expression was calculated using the 2^−ΔΔCT^ method, with β‐actin as an internal reference. The following PCR primers were used: for β‐actin, forward: 5′‐TATCCTGGCCTCACTGTCCA‐3′, reverse: 5′‐AAGGGTGTAAAACGCAGCTC‐3′; for Arg1, forward: 5′‐TCATGGAAGTGAACCCAACTCTTG‐3′, reverse: 5′‐TCAGTCCCTGGCTTATGGTTACC‐3′; for CD206, forward: 5′‐GGTTCCGGTTTGTGGAGCAG‐3′, reverse: 5′‐TCCGTTTGCATTGCCCAGTA‐3′.

### 2.12 Animal Experiments

Healthy male Sprague–Dawley rats (240–260 g) were purchased from Jinan Pengyue Laboratory Animal Breeding Co. Ltd., China [License no.: SCXK (Lu) 20220006]. A total of 36 rats were split into four groups at random: Sham, Glioma, Glioma + ML130, and Glioma + ie‐DAP (*n* = 9 per group). The rats were anesthetized, and an in situ glioma model was established using a stereotaxic device to implant C6 glioma cells (approximately 5 × 10^5^ cells/10 μL) into the right striatum of the rats. Rats in the Glioma, Glioma + ML130, and Glioma + ie‐DAP groups underwent glioma model establishment, whereas those in the Sham group were injected with an equivalent volume of complete medium. All rats were fed at room temperature (21°C ± 2°C) in alternating light and dark conditions. ML130 and ie‐DAP were diluted with saline after being dissolved in DMSO. In order to optimize the dose of ML130 specifically for glioma, different doses of ML130, including 0.5, 1, and 2 mg/kg, were administered every other day in the preliminary trial. Rats in the Glioma + ML130 and Glioma + ie‐DAP groups received intraperitoneal injections of ML130 (1 mg/kg; MCE) and ie‐DAP (3 mg/kg; MCE) starting on the 7th day after model establishment and continuing every other day [26] until the 14th day. Meanwhile, the Sham and Glioma groups received equal amounts of DMSO‐containing saline during the same period [23]. This experiment was approved by the Animal Ethics Committee of the Second Medical University of Shandong Province (approval no.: 2024SDL871).

### 2.13 MRI Data Acquisition

To monitor glioma growth using T2WI, a 7.0 T MRI scanner (BioSpec 70/20; Bruker, Germany) was used to scan rat brain tissue on the 7th and 14th days after glioma cell injection. The main scan parameters were as follows: 29 slices, repetition time (TR) = 3000 ms, echo time (TE) = 24 ms, field of view (FOV) = 33 mm × 31 mm, slice thickness = 0.5 mm, and matrix = 160 × 150. T2WI images were processed using 3D‐Slicer software (version 4.11.0, available at www.slicer.org). We first manually delineated the tumor boundaries on each slice containing the tumor, and then the area of the tumor on each image was calculated in the software. The area of the image was then multiplied by the slice thickness to compute a volume, and the volumes of each image were summarized to calculate the tumor volume, which was used to calculate the tumor growth rate. The brain volume was obtained using the same method. The formula for calculating tumor growth rate is Tumor growth rate = (14 days tumor volume/14 days brain volume − 7 days tumor volume/7 days brain volume)/(7 days tumor volume/7 days brain volume).

### 2.14 Hematoxylin and Eosin Staining

A hematoxylin solution was used to stain the nuclei in brain tissue sections, followed by an eosin solution for staining the cytoplasm. The sections were sealed with neutral gum, and pathologic changes in the brain tissue were observed using an optical microscope (BX51; Olympus).

### 2.15 Immunohistochemical Staining

The tissue sections were dewaxed, hydrated, and subjected to antigen retrieval. The sections were then incubated with the following antibodies: NOD1 (1:200; Affinity), RIP2 (1:200; Affinity), Iba1 (1:1000; Yeasen), rabbit anti‐interleukin‐1β (IL‐1β) (1:1000; Affinity), and Mannose receptor C type 1 (1:1000; MCE). Immunohistochemical staining was performed using a kit (PV‐9001; ZSGB‐Bio). The sections were sealed with neutral gum, and the expression of markers was observed using an optical microscope (BX 51; Olympus).

### 2.16 Western Blot Analysis

Fresh brain glioma tissues were collected from each group 14 days after glioma cell injection. Proteins from the brain and cells were isolated using a radioimmunoprecipitation assay buffer (CR2302079; Servicebio). The supernatant was collected after high‐speed centrifugation, and the protein concentration was measured using a Bicinchoninic Acid kit (KGP902; KeyGEN, China). The proteins were separated through 12% sodium dodecyl sulfate–polyacrylamide gel electrophoresis and transferred onto polyvinylidene fluoride (PVDF) membranes. The membranes were incubated overnight with primary antibodies: glyceraldehyde‐3‐phosphate dehydrogenase (1:100,000; Servicebio), NOD1 (1:1000; Affinity), RIP2 (1:1000; Affinity), Iba1 (1:1000; Yeasen), IL‐1β (1:1000; Affinity), and MRC1 (1:1000; MCE). The following day, PVDF membranes were incubated with secondary antibodies. Images were captured using an imaging system (FluorChem HD2; Protein Simple, USA) after luminescence, and optical density (OD) values were measured using ImageJ software.

### 2.17 Statistical Analysis

GraphPad Prism 9.5 was used to analyze the data. All data were verified for normal distribution by the Shapiro–Wilk test and presented as mean ± standard deviation. An independent‐samples *t* test was used for comparing two experimental groups, whereas one‐way analysis of variance was used for comparing data across several groups. The correlation between NOD1/RIP2 and microglial polarization was analyzed using Pearson's correlation.

## 3 Results

### 3.1 Expression and Imaging Markers of NOD1 in Gliomas Across Varying Grades

Samples from the CGGA database were analyzed to determine the expression characteristics of NOD1. The results demonstrated that NOD1 expression varied between tumor and normal tissues (Figure S1A). High NOD1 expression was associated with several clinical features, particularly World Health Organization (WHO) grades (Figure S1B,C,E) and glioma survival times (Figure S1D). Additionally, NOD1 expression was strongly correlated with macrophage and microglia presence (Figure S1F). According to WB analysis, NOD1 expression was significantly lower in non‐tumor tissues and significantly higher in high‐grade gliomas than that in low‐grade gliomas (Figure S1G,H) (*p* < 0.05).

**FIGURE 1:**
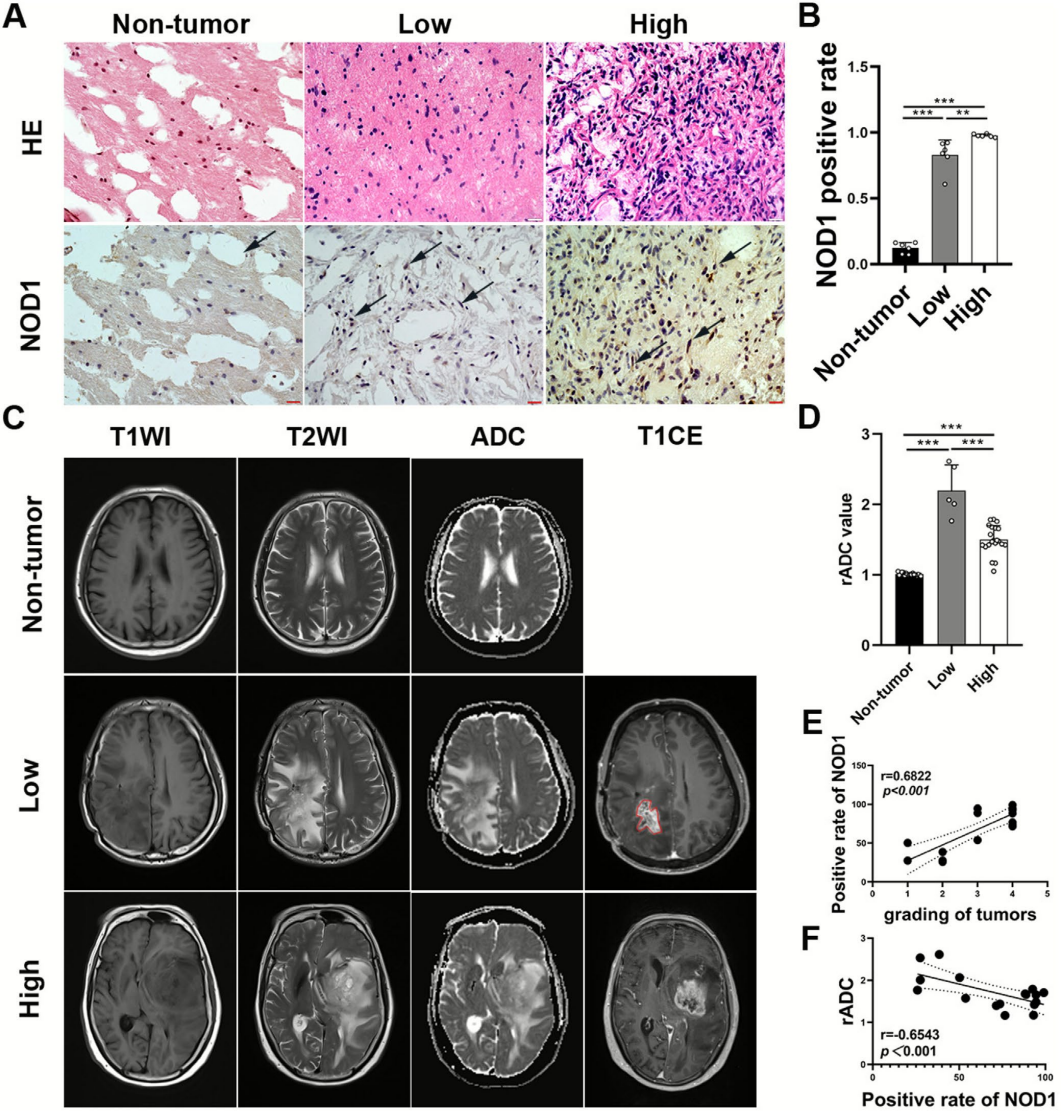
Imaging markers of NOD1 in glioma. (A) Representative images of HE and IHC staining showing NOD1 expression in brain tissues of each group (scale bar = 20 μm). (B) Comparison of the proportion of NOD1‐positive cells in brain tissues of each group (*n* = 6). (C) Representative MRI images of each group. (D) Comparison of rADC values between groups. (E) Correlation analysis of NOD1 expression with glioma grade. (F) Correlation analysis of NOD1 expression with rADC values. Data are presented as mean ± standard deviation. **p < 0.01 and ***p < 0.001.

As observed in the HE and immunohistochemical (IHC) images (Figure 1A), the nuclei in non‐tumor tissues exhibited regular morphology and were sparsely arranged. In contrast, tissues from the high‐grade glioma group exhibited increased nuclear density, irregular nuclear morphology, and numerous dividing nuclei. IHC analysis revealed more NOD1‐positive cells in glioma tissues than in non‐tumor tissues, with high‐grade gliomas having a markedly greater proportion of NOD1‐positive cells than low‐grade gliomas (*p* < 0.01, Figure 1B). MRI imaging demonstrated distinct patterns in each group. Non‐tumor tissues displayed signs of lacunar infarction of brain tissues. In contrast, glioma tissues exhibited an intracranial low‐signal occupying area on T1WI, an irregular high‐signal area with peripheral edema on T2WI, and restricted water molecule dispersion in the tumor area on ADC images. T1CE revealed intracranial high‐signal enhancement areas and vascular enhancement signal shadows in patients with glioma (Figure 1C). The ADC values reflect water molecule movement in vivo [27]. The low‐grade glioma group had significantly higher relative ADC (rADC) values than the non‐tumor group, while the high‐grade glioma group had lower rADC values than the low‐grade group (Figure 1D). Correlation analysis demonstrated that NOD1 expression correlated positively with glioma grade and negatively with rADC values (Figure 1E,F).

### 3.2 NOD1/RIP2 Pathway Was Associated With Microglial M2 Polarization

**FIGURE 2:**
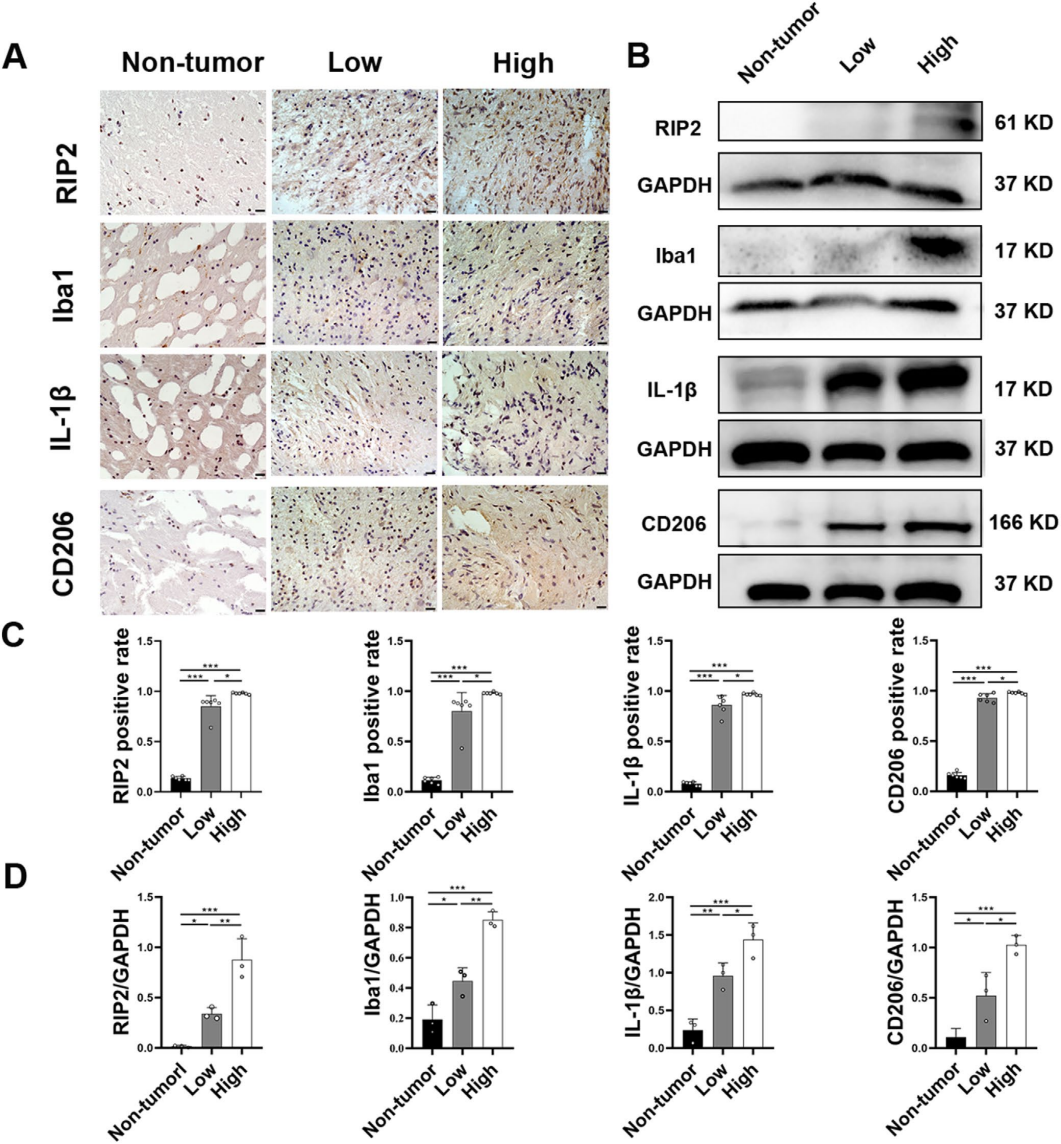
NOD1/RIP2 correlated with microglial polarization in human glioma tissues. (A) IHC results for RIP2, Iba1, IL‐1β, and CD206 in brain tissues from each group (scale bar = 20 μm). (B) WB results for RIP2, Iba1, IL‐1β, and CD206 proteins in each group. (C) Proportion of positive cells for RIP2, Iba1, IL‐1β, and CD206 in rats from each group (*n* = 6). (D) Comparison of RIP2, Iba1, IL‐1β, and CD206 protein expression levels in rats from each group (*n* = 3). *p < 0.05, **p < 0.01, and ***p < 0.001.

Iba1 is a marker of microglia [28]. Arg1 and CD206 are markers of M2 microglia [29], whereas IL‐1β is a marker of M1 microglia [30]. According to Figure 2A,C, glioma tissues had a higher percentage of positive cells for RIP2, Iba1, IL‐1β, and CD206 than non‐tumor tissues (*p* < 0.05), and the high‐grade glioma group had higher expression of each signal than the low‐grade glioma group (*p* < 0.05). The WB result (Figure 2B,D) indicated that the relative expression of RIP2, Iba1, IL‐1β, and CD206 increased in glioma tissues, with higher expression in high‐grade gliomas than in low‐grade gliomas (*p* < 0.05). Correlation analysis of NOD1 and RIP2 with Iba1 and CD206 expression (Figure S2A) revealed positive correlations between them.

### 3.3 Targeted Inhibition of NOD1 Inhibited Proliferation, Migration, and Invasion of Glioma Cells

**FIGURE 3:**
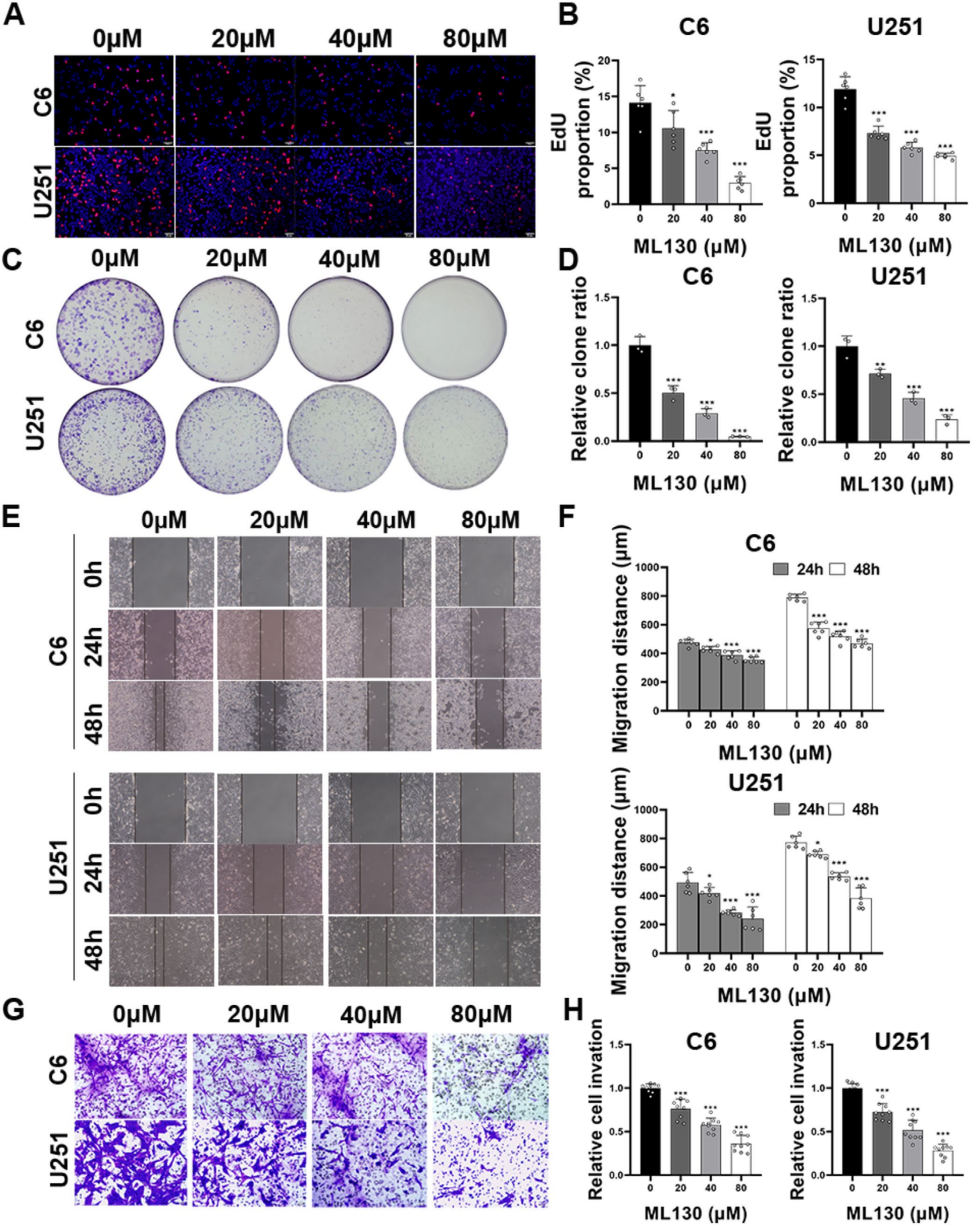
Invasion, migration, and proliferation of glioma cells were suppressed by ML130. (A) Representative EdU assay images of two cell lines (scale bar = 50 μm). (B) Statistical results of the EdU assay. (C) Representative plate cloning assay images of two cell lines. (D) Statistical results of the plate cloning assay. (E) Representative wound healing assay images of two cell lines. (F) Statistical results of the wound healing assay. (G) Representative Transwell invasion assay images of two cell lines. (H) Statistical results of the Transwell invasion assay. *p < 0.05, **p < 0.01, and ***p < 0.001.

The cell activity of ML130 (0, 10, 20, 40, 60, and 80 μM), GSK‐583 (0, 5, 10, and 20 μM), and ie‐GAP (0, 15, 30, 60, 90, and 120 μM) was detected by the CCK‐8 method. ML130 and GSK‐583 began to show significant inhibitory effects at 20 and 10 μM respectively, while ie‐GAP had no cytotoxic effect at 30 μM (*p* < 0.001, Figure S2B–D). Therefore, four concentration gradients (0, 20, 40, and 80 μM) were used in subsequent experiments, 10 μM was used for GSK‐583, and 30 μM was used for ie‐GAP. WB and IF analyses revealed that the expression of NOD1 and RIP2 proteins decreased with the increase in ML130 concentration (*p* < 0.05) (Figure S2E–H). EdU and plate cloning assays demonstrated that ML130 inhibited the proliferation of C6 and U251 cells, with proliferative capacity decreasing with the increase in ML130 concentration (Figure 3A–D). Wound healing assay results demonstrated substantially reduced migration efficiency of glioma cells after 24 and 48 h of ML130 treatment (Figure 3E,F). Transwell invasion experiment results indicated reduced in vitro invasiveness of glioma cells with increasing ML130 concentration (Figure 3G,H).

### 3.4 Microglial M2 Polarization Was Regulated by the NOD1/RIP2 Pathway, Promoting the Biologic Behavior of Glioma Cells

**FIGURE 4:**
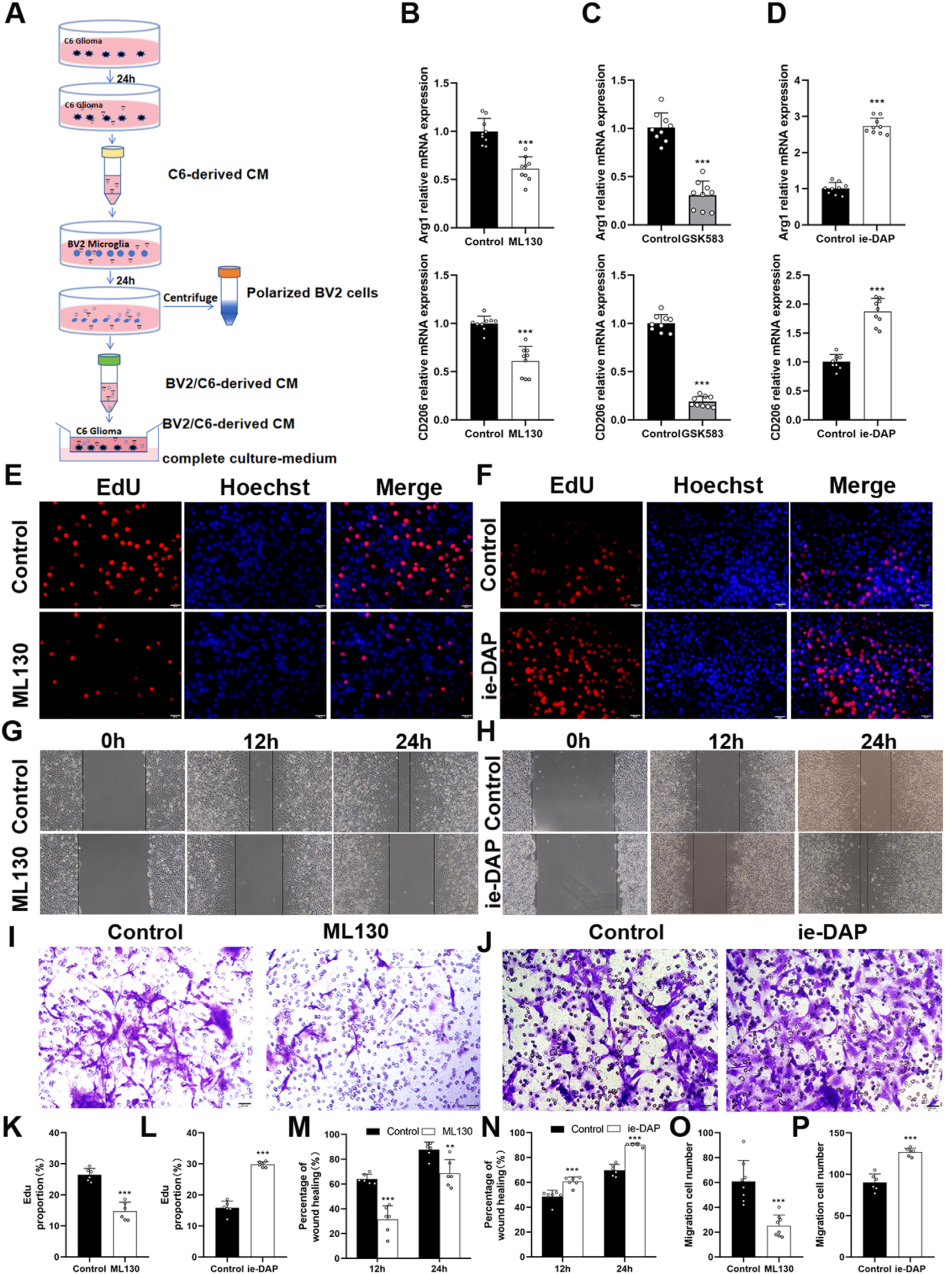
Microglial polarization was regulated through the NOD1/RIP2 pathway, influencing glioma cell behavior. (A) Schematic diagram of C6‐derived CM and C6/BV2‐derived CM collection and treatment. (B) qRT‐PCR results of Arg1 and CD206 in CM cultured BV2 cells pretreated with ML130. (C) qRT‐PCR results of Arg1 and CD206 in CM cultured BV2 cells pretreated with GSK‐583. (D) qRT‐PCR results of Arg1 and CD206 in CM cultured BV2 cells pretreated with ie‐GAP. (E, F) Representative EdU assay images of C6 cells (scale bar = 50 μm). (G, H) Representative wound healing assay images of C6 cells. (I, J) Representative Transwell assay images of C6 cells treated with C6/BV2‐derived CM. (K, L) Statistical results of the EdU assay. (M, N) Statistical results of wound healing assay. (O, P) Statistical results of the Transwell assay. **p < 0.01 and ***p < 0.001.

C6‐derived and C6/BV2‐derived CMs were collected and used to treat BV2 and C6 cells, respectively (Figure 4A). qRT‐PCR analysis showed that the expression levels of Arg1 and CD206 in BV2 cells in both ML130 and GSK‐583 groups were significantly lower than those in the control group (*p* < 0.001) (Figure 4B,C). The expression levels of Arg1 and CD206 in BV2 cells of the ie‐GAP group were significantly higher than those of the control group (*p* < 0.001) (Figure 4D). Meanwhile, C6 cells were cultured with C6/BV2‐derived CM, and the effects of ML130 and ie‐GAP on the proliferation, migration, and invasion of glioma cells were assessed using EdU, wound healing, and Transwell assays (Figure 4E–P). The results showed that compared with the control group, the proliferation, migration, and invasivity of C6 cells in the ML130 treatment group significantly decreased (*p* < 0.01), while those in the ie‐GAP treatment group increased (*p* < 0.01). Furthermore, the underlying mechanisms were investigated using combinations of GSK‐583 with ML130 and ie‐DAP. Compared with the control group, the expression of Arg1 and CD206 in microglia in both GSK‐583 + ML130 and GSK‐583 + ie‐DAP groups was significantly inhibited, and the inhibitory effect was more significant in the GSK‐583 + ML130 group (*p* < 0.05) (Figure S3A). Additionally, the proliferation, migration, and invasion abilities of C6 cells were significantly reduced in both GSK‐583 + ML130 and GSK‐583 + ie‐DAP groups, with a more pronounced decrease observed in the GSK‐583 + ML130 group (Figure S3B–G).

### 3.5 Microglial Polarization Was Regulated by the NOD1/RIP2 Pathway In Vivo

**FIGURE 5:**
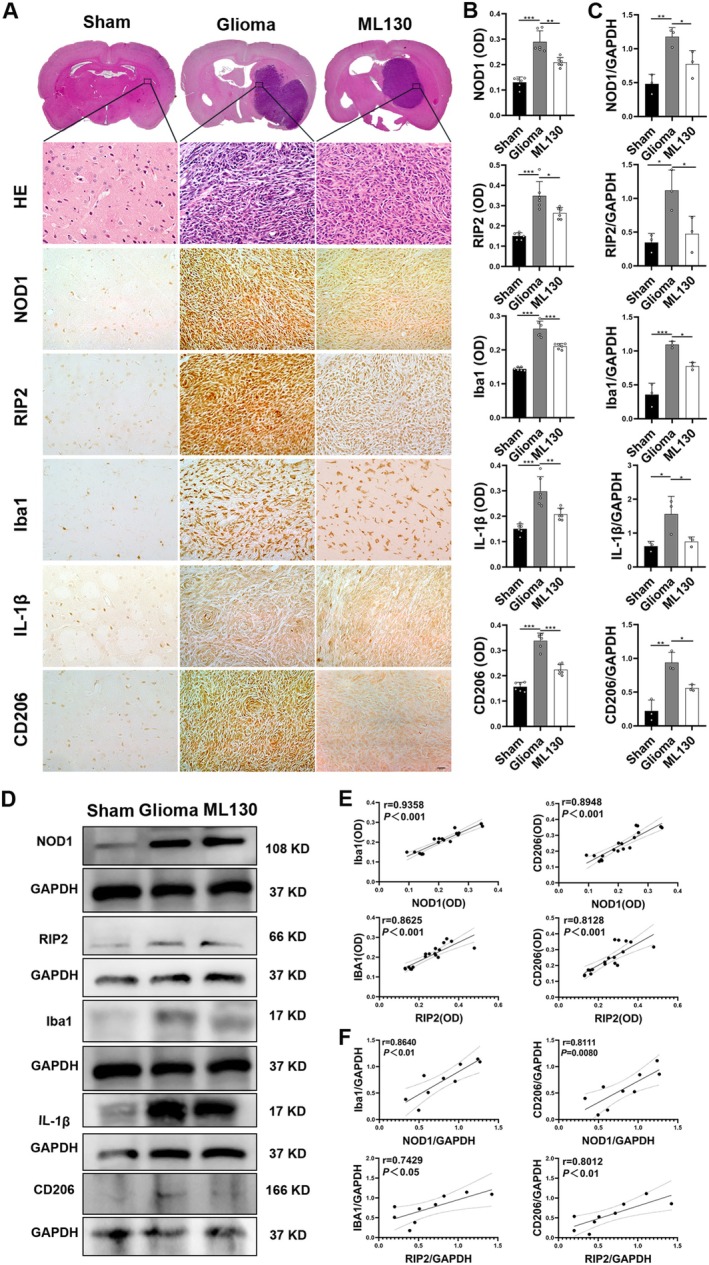
Microglial polarization in the rat brain was regulated through the NOD1/RIP2 pathway. (A) HE and IHC results for NOD1, RIP2, Iba1, IL‐1β, and CD206 in brain tissues of each group (scale bar = 20 μm). (B) OD values for NOD1, RIP2, Iba1, IL‐1β, and CD206 proteins in rats of each group (*n* = 6). (C) Comparison of relative expression levels of NOD1, RIP2, Iba1, IL‐1β, and CD206 proteins in rats of each group (*n* = 3). (D) WB results for NOD1, RIP2, Iba1, IL‐1β, and CD206 proteins in rats of each group. (E) Correlation analysis of OD values for NOD1 and RIP2 with Iba1 and CD206. (F) Correlation analysis of relative expression of NOD1 and RIP2 with Iba1 and CD206. *p < 0.05, **p < 0.01, and ***p < 0.001.

A glioma model was established, and brain tissues were collected for HE and IHC staining. HE staining (Figure 5A,B) revealed homogeneous morphology and regular arrangement of cells in the Sham group, whereas glioma tissues exhibited increased cell numbers, nuclear heterogeneity, and more dividing nuclei. Compared with the Glioma group, the number of cells in the brain tissue of the Glioma + ML130 group was lower. The IHC results showed that in the Glioma + ML130 group, the OD values of NOD1, RIP2, Iba1, IL‐1β, and CD206 were lower than those in the Glioma group (*p* < 0.05). WB results (Figure 5C,D) exhibited relative protein expression levels of NOD1, RIP2, Iba1, IL‐1β, and CD206 in glioma tissues in the Glioma group that were higher than in the Sham group (*p* < 0.05), with decreased expression following ML130 treatment. As revealed in Figure 5E,F, the correlation between the expression levels of NOD1 and RIP2 with those of Iba1 and CD206 was analyzed. The findings showed that NOD1 expression was positively correlated with Iba1, IL‐1β, and CD206 expression levels.

### 3.6 Inhibition of NOD1 Expression Reduced the Glioma Growth Rate

**FIGURE 6:**
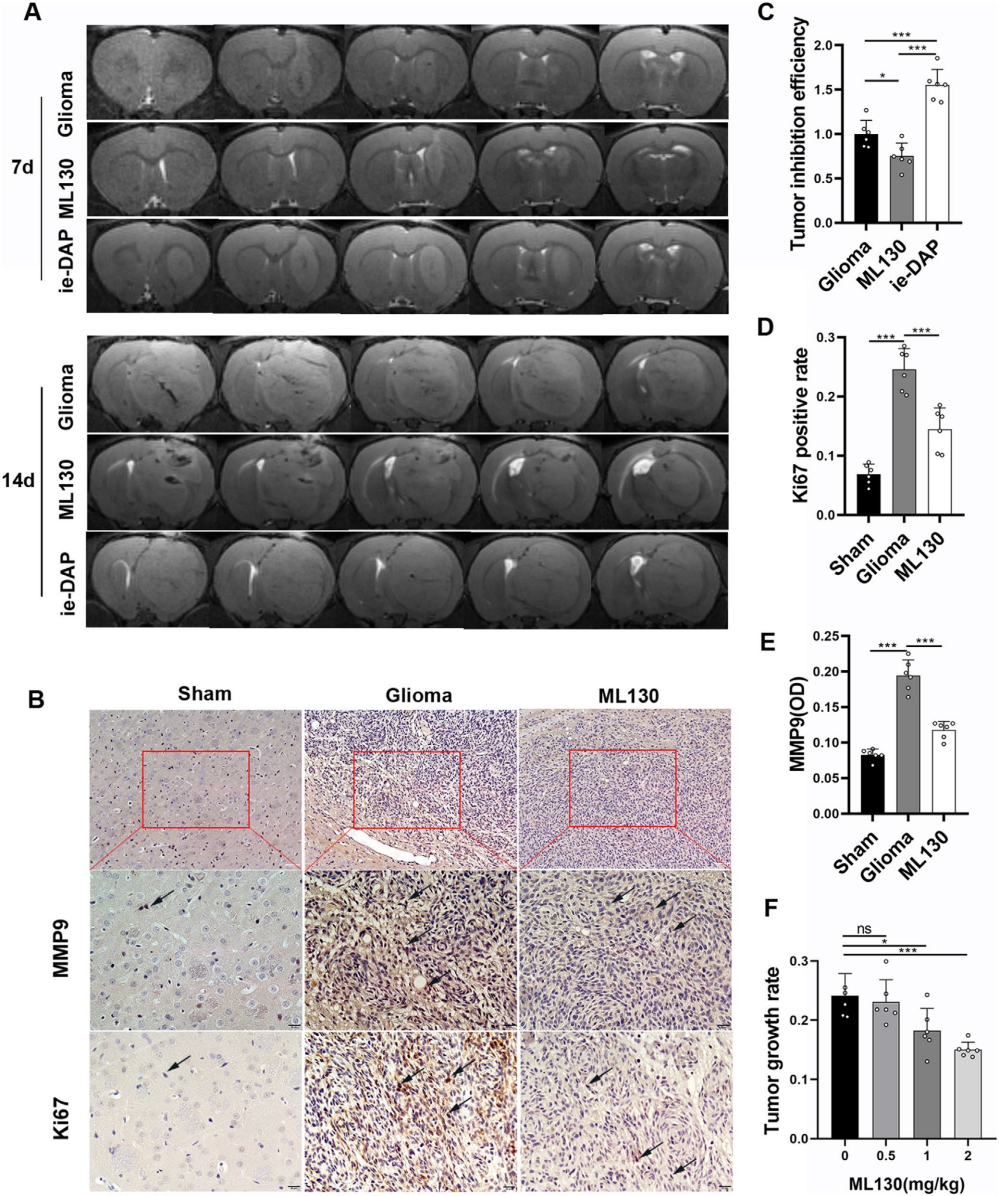
ML130 inhibited glioma cell proliferation. (A) T2WI images of glioma rats on Days 7 and 14. (B) Representative IHC images of MMP9 and Ki67 in rat brain tissues of each group (scale bar = 20 μm) (*n* = 6). (C) Comparison of glioma growth rates in the Glioma and Glioma + ML130 groups (*n* = 3). (D) Comparison of the expression of Ki67 in rat brain tissue of each group (*n* = 6). (E) Comparison of the MMP9 expression in rat brain tissues across the three groups (*n* = 6). (F) The effects of different concentrations of ML130 (*n* = 3). *p < 0.05, ***p < 0.001.

An in situ glioma model was established to evaluate the effects of different doses of ML130 on tumor growth. The results showed that 1 mg/kg of ML130 had an inhibitory effect on tumors (Figure 6F). The MRI scan results indicated that ML130 significantly inhibited the intracranial growth rate of rat gliomas, while ie‐DAP promoted the growth rate (Figure 6A,C). IHC analysis of rat brain tissues revealed that the expression levels of Ki67 (a marker of cell proliferation) and matrix metalloproteinase‐9 (MMP9, an indicator of cell invasion ability) were significantly higher in the Glioma group than in the Sham group (*p* < 0.001). However, ML130 treatment substantially reduced the expression of these markers, as demonstrated in Figure 6B,D,E.

## 4 Discussion

The prognosis is dismal for gliomas and gliomas are extremely aggressive malignant tumors of the central nervous system. Traditional treatments have limited effectiveness in improving outcomes for patients [31]. Enhancing glioma prognosis requires uncovering the molecular mechanisms underlying its development and exploring novel therapeutic strategies. Previous studies have demonstrated that NOD1 is abnormally expressed in various tumors and can play a role in cancer diagnosis [32], monitoring [18], resistance [24], and prognosis [33]. Bioinformatics analysis using data from the CGGA database showed that the expression of NOD1 in glioma was associated with several clinical features, particularly tumor grading. This finding was further supported by a WB analysis, which revealed NOD1 expression in glioma tissues of varying grades. Thus, NOD1 can serve as a biomarker for glioma diagnosis and a target for treatment. NOD1 expression was considerably higher in human glioma tissues and positively correlated with glioma malignancy. rADC is a post‐processing constant derived from DWI‐MRI sequences and has been considered an indicator of the diffusion ability of water molecules in vivo.

rADC has been reported to be negatively correlated with cell proliferation ability [34]. This study demonstrated that rADC was negatively associated with glioma grade, aligning with previous research [35]. In addition, this study revealed that NOD1 expression was negatively correlated with rADC values, suggesting that MRI could serve as an imaging marker for predicting NOD1 expression. The high NOD1 expression may therefore indicate malignant features in glioma, although the precise mechanism of NOD1 in glioma progression remains unclear.

Bioinformatics analysis in this study also revealed a correlation between NOD1 expression and infiltration of various immune cells, especially macrophages (microglia), consistent with the results reported in previous studies [36, 37]. The mechanism of microglial M2 polarization during glioma has been well documented [38, 39]. This suggests that the NOD1/RIP2 pathway may regulate microglial M2 polarization, thereby influencing glioma growth. The relationship between the NOD1/RIP2 pathway and microglial polarization was investigated by performing IHC and WB analyses on human non‐tumor brain and glioma tissues. The results indicated that NOD1, RIP2, Iba1, IL‐1β, and CD206 were highly expressed in glioma tissues, with higher expression in high‐grade gliomas than in low‐grade gliomas. Furthermore, a positive correlation was observed between the expression of Iba1 and CD206 and the expression of NOD1 and RIP2. These findings suggest that microglial M2 polarization may be regulated by the NOD1/RIP2 pathway, influencing glioma progression.

To further explore the effects of NOD1 on glioma cell behavior, the NOD1 inhibitor ML130 was used to culture two types of glioma cells: C6 and U251. A CCK‐8 assay determined the optimal concentration of ML130, revealing marked inhibition of cell activity at 20 μM, with a notable concentration‐dependent effect. The EdU, plate cloning, wound healing, and Transwell invasion assays demonstrated that ML130 inhibited glioma cell migration, proliferation, and invasiveness. RIP2, a downstream signaling molecule of NOD1, has been implicated in the migration and invasion of various tumors [25, 40]. The expression of RIP2 in ML130‐treated glioma cells was explored to elucidate the effect of NOD1 on the downstream signaling molecule RIP2. The results revealed that NOD1 and RIP2 expression decreased with increasing ML130 concentration. This indicated that ML130 suppressed NOD1 expression, subsequently inhibiting the production of its downstream signaling component RIP2. Although the NOD1/RIP2 pathway has been implicated in various inflammatory responses [21, 41], its role in glioma remains unclear. To further elucidate how the NOD1/RIP2 pathway regulates microglial M2 polarization and affects glioma progression, C6‐derived and C6/BV2‐derived CMs were collected and used to culture BV2 and C6 cells, respectively. The results of qRT‐PCR showed that the expression levels of Arg1 and CD206 in BV2 cells of the ML130 group, and the GSK‐587 group were lower than those of the control group, while the expression in the ie‐GAP group increased, indicating that the NOD1/RIP2 pathway affected the M2 polarization of microglia. Additionally, EdU, wound healing, and Transwell demonstrated that compared with the control group, the proliferation, migration and invasion abilities of C6 cells in the ML130 group decreased while increased in the ie‐GAP group. To further verify that NOD1 affects microglial polarization through RIP2, a coculture experiment was conducted by treating the cells with GSK‐583 + ML130 and GSK‐583 + ie‐DAP. The results indicate that NOD1 can promote microglial cell polarization through RIP2, thereby influencing the biological behavior of glioma cells. These findings suggest that the NOD1/RIP2 signaling pathway regulates microglial M2 polarization, affecting the proliferation and invasion ability of glioma cells.

An in situ glioma model was established to confirm the impact of the NOD1/RIP2 pathway on microglial polarization in vivo. WB and IHC assays were used to examine the NOD1/RIP2 expression and microglial polarization markers in rat brain tissues. Compared with the Glioma group, the expressions of NOD1, RIP2, Iba1, IL‐1β, and CD206 in the Glioma + ML130 group were all decreased. Correlation analysis further confirmed a positive correlation of NOD1 and RIP2 expression with Iba1 and CD206 levels. Thus, the NOD1/RIP2 pathway influences microglial polarization in vivo. The effects of ML130 and ie‐DAP on glioma growth rates in rats were analyzed to assess whether the NOD1/RIP2 pathway could influence glioma progression. The growth rate of glioma in the Glioma + ML130 group was inhibited, while the growth rate in the Glioma + ie‐DAP group increased. Ki67 and MMP9, markers of glioma proliferation [42] and invasiveness [8], respectively, demonstrated higher expression in the Glioma group than in the Glioma + ML130 group, as evidenced by IHC. These results suggest that the NOD1/RIP2 pathway is crucial for in situ glioma invasiveness and proliferation in rats.

This study highlighted the essential role of NOD1 in glioma progression and its positive correlation with WHO grades. The rADC value was negatively correlated with NOD1 expression in the brain tissues of patients with glioma, providing an imaging biomarker for predicting the NOD1 expression. NOD1 influences microglial M2 polarization by regulating the NOD1/RIP2 pathway, thereby promoting glioma progression. Targeting the NOD1/RIP2 pathway effectively inhibits glioma progression, offering a theoretical basis for precise and individualized glioma treatment.

## Author Contributions

Yuxue Luan and Miao Yu participated in all the experiments and wrote the first draft. Haimo Zhang, Xiaozu Zhang, and Tianyu Zhao conducted part of the experiments. Fenghua Zhou and Peilun Xiao provided meaningful suggestions for the project. Xiaoli Wang and Xizhen Wang supervised the project and revised the manuscript. The final manuscript was reviewed and approved by all authors.

## Funding

This work was supported by the National Natural Science Foundation of China (grant number 82071888), Shandong Provincial Natural Science Foundation (grant number ZR2024MH067), Weifang Science and Technology Development Project (grant number 2021GX058 and 2021YX062), and Science and Technology Development Project of the Affiliated Hospital of Shandong Second Medical University (grant number 2024FYZ004, 2024FYQ005, and 2024FYM021).

## Ethics Statement

The experiment was approved by the Medical Ethics Committee of Shandong Second Medical University (approval number: 2022YX048) and the Animal Ethics Committee of the Second Medical University of Shandong Province (approval number: 2024SDL871).

## Conflicts of Interest

The authors declare no conflicts of interest.

## Supporting information


**Figures S1–S3:** cns70674‐sup‐0001‐FiguresS1‐S3.docx.

## Data Availability

The data that support the findings of this study are available from the corresponding author upon reasonable request. The data are not publicly available due to privacy or ethical restrictions.
