# Supplementary material for: NOD1/RIP2 Pathway Promotes Glioma Progression Through Microglial M2 Polarization
Source: CNS Neurosci Ther. 2025 Dec 9;31(12):e70674. doi: 10.1111/cns.70674 (PMC12686968; doi:10.1111/cns.70674)
Supplement: Supplementary file 1 — Figures S1–S3: cns70674‐sup‐0001‐FiguresS1‐S3.docx. [file CNS-31-e70674-s001.docx]

**Figure S1 Expression characteristics and bioinformatics analysis of NOD1 in glioma** (A) Pan-cancer analysis of NOD1 differential expression in multiple tumour tissues. (B) NOD1 is differentially expressed in normal brain tissues versus s. (D) Survival curve analysis of the relationship between NOD1 exprglioma tissues. (C) Differential expression of NOD1 in different graded glioma tissueession and patients' overall survival (OS). (E) The correlation between NOD1 expression and the clinical manifestations of glioma patients. (F) Immune infiltration analysis showing the correlation between NOD1 expression and multiple immune cells. (G) Western Blots results of NOD1 protein expression in non-tumor group, high and low grade glioma tissues. (H) Comparison of NOD1 protein expression in brain tissues of each group (n = 3). Results are presented as mean ± SD. *^*^P* < 0.05, *^**^P* < 0.01, *^***^P* < 0.001.

**Figure S2 ML130 affected the expression of key proteins (NOD1 and RIP2) in the NOD1/RIP2 signaling pathway in glioma cells.** (A) Correlation analysis of NOD1/RIP2 pathway and polarization protein expression in microglia in human glioma tissues. (B) The influence of different concentrations of ML130 on the cell viability of glioma cells. (C) The effects of different concentrations of GSK-583 on the cell viability of C6 cells. (D) The effects of different concentrations of ie-DAP on the cell viability of C6 cells. (E) Representative pictures of Western Blots of NOD1, RIP2 protein expression after different concentrations of ML130 treatment. (F) Representative pictures of NOD1 and RIP2 immunofluorescence staining after different concentrations of ML130 treatment (scale bar = 50µm). (G) Statistics of Western Blots results (n = 3). (H) Statistics of the average fluorescence intensity of immunofluorescence staining.Results are presented as mean ± SD. *^*^P* < 0.05, *^**^P* < 0.01, *^***^P* < 0.001, ns, not significant.

**Figure S3 The role of the NOD1/RIP2 pathway in microglial polarization and glioma cell biology.** (A) qRT-PCR results of Arg1 and CD206 in CM cultured BV2 cells. (B) Representative EdU assay images of C6 cells (scale bar = 50 µm). (C) Statistical results of the EdU assay. (D) Representative Transwell assay images of C6 cells treated with C6/BV2-derived (E) Statistical results of the Transwell assay. (F) Representative wound healing assay images of C6 cells. (G) Statistical results of wound healing assay. Results are presented as mean ± SD. *^*^P* < 0.05, *^**^P* < 0.01, *^***^P* < 0.001, ns, not significant.
